# Exploring the Impact of Digital Inclusive Finance on Agricultural Carbon Emission Performance in China

**DOI:** 10.3390/ijerph191710922

**Published:** 2022-09-01

**Authors:** Le Sun, Congmou Zhu, Shaofeng Yuan, Lixia Yang, Shan He, Wuyan Li

**Affiliations:** 1 School of Finance, Zhejiang Gongshang University, Hangzhou 310018, China; 2 School of Humanities and Art Design, Zhejiang Gongshang University Hangzhou College of Commerce, Hangzhou 310018, China; 3 Department of Land Resources Management, Zhejiang Gongshang University, Hangzhou 310018, China; 4 School of Public Administration, Zhejiang University of Finance and Economics, Hangzhou 310018, China; 5 College of Economics and Management, China Jiliang University, Hangzhou 310018, China; 6 The Institute of Land and Urban-Rural Development, Zhejiang University of Finance and Economics, Hangzhou 310018, China

**Keywords:** agricultural carbon emission performance, digital inclusive finance, super SBM model, panel regression model, spatial regression model, China

## Abstract

This paper attempts to reveal the impact and mechanisms of digital inclusive finance (DIF) on agricultural carbon emission performance (ACEP). Specifically, based on the provincial panel data in China from 2011 to 2020, a super slacks-based measure (Super SBM) model is applied to measure ACEP. The panel regression model and spatial regression model are used to empirically analyze the impact of DIF on ACEP and its mechanism. The results show that: (1) during the study period, China’s ACEP exhibited a continuous growth trend, and began to accelerate after 2017. The high-value agglomeration areas of ACEP shifted from the Huang-Huai-Hai plain and the Pearl River Delta to the coastal regions and the Yellow River basin, the provincial differences displayed an increasing trend from 2011 to 2020. (2) DIF was found to have a significant positive impact on ACEP. The main manifestation is that the development of the coverage breadth and depth of use of DIF helps to improve the ACEP. (3) The positive impact of DIF on ACEP had a significant spatial spillover effect, that is, it had a positive effect on the improvement of ACEP in the surrounding provinces. These empirical results can help policymakers better understand the contribution of DIF to low-carbon agriculture, and provide them with valuable information for the formulation of supportive policies.

## 1. Introduction

As the Gloabl-warming effect intensifies, accelerating low-carbon development has become an important development strategy for various countries around the world [1]. In 2020, Chinese government formally set out strategic goals for peaking carbon emissions by 2030 and achieving carbon neutrality by 2060. This ambitious goal, which can significantly slow global warming, drives China’s development toward a low-carbon economy [2]. In China, agriculture is a major source of carbon emissions, accounting for 17% of the country’s total carbon emissions, significantly higher than the global average of 11% [3]. Promoting carbon emission reduction in agriculture is not only an important part of China’s “dual carbon” goal, but also an indispensable content of accelerating the construction of agricultural ecological civilization. Therefore, To reduce carbon emissions, one of China’s main approach is to improve the agricultural carbon emission performance (ACEP), which can reflect the agricultural factor productivity resourced from multiple factors such as agricultural material input, human consumption and economic develop [4,5]. Finance plays a key role in the resource allocation of agricultural production, and thus has a significant impact on the productivity of the agricultural sector [6,7]. Due to the high financing costs and inefficiencies, traditional finance struggles to support technological innovation and green development in agriculture [8]. Emerging digital inclusive finance (DIF) can overcome the challenges that traditional finance faces. It is a new financial service model to improve financial inclusion by using digitial technologies and the Internet [9,10]. In recent years, China’s world-leading DIF is reshaping the way of production and life. Therefore, examining the impact of DIF on ACEP is of great significance to promoting low-carbon agriculture and achieving China’s carbon-reduction goals.

ACEP is regarded as an important metric when evaluating the low-carbon development of agriculture [11]. The method of calculating ACEP is attracting increasing attention from domestic and foreign scholars. At present, two most credible approaches, including the stochastic frontier analysis (SFA) and the data envelopment analysis (DEA) are widely used [12,13]. The SFA method takes the production cost, the production function, and the mixed error term into account. But detractors of the method note the need to specify the distribution of the random error term. They also point to the fact that frontier function is susceptible to spatio-temporal differences [14]. Looking for a calculation that would ensure the robustness of the time trend, Chen and Gong (2021) applied four different production functions to measure agricultural total factor productivity in China [15]. Compared with the SFA method, the DEA method does not need a predetermined specific production function. Furthermore, it measures the factor productivity level by comparing it with observed best practice results [13]. Thus, it is more suitable for frontier production functions with multiple inputs and multiple outputs. This method is closely related to concepts such as total factor productivity and green productivity [16,17]. Given these factors, it has become the main approach for measuring ACEP. However, the traditional DEA method cannot accurately calculate actual efficiency because agricultural production is easily affected by external environmental factors and stochastic disturbances [7]. To overcome these shortcomings, many scholars have adopted the improved slacks-based measure (SBM) model proposed by Tone (2001) [18]. By basing the SBM model on relaxation variables, it can accurately measure total factor productivity [13,19]. To further evaluate and rank the effectiveness of the decision units, a Super SBM model based on modified relaxation variables was later proposed and is now widely used [7,14]. This paper attempts to calculate the ACEP by using the Super SBM model.

The continuous improvement of ACEP assessment methods has increased the wider discussion around the influencing factors of ACEP. Urbanization [14], industrial structure [20], pure technological advancement [21], public infrastructure and human capital [22], were proven to be beneficial for ACEP improvement. Further factors include the agricultural technology and agricultural industrial structure [23]. In addition, Chen et al. (2018) found that there was a Kuznets curve interactive relationship between agro-industrial agglomeration and ACEP [24]. One factor of particular interest is finance development, as it provides necessary funding for technological innovation and essential investments for economic growth, affecting the economic development model and the environment [25]. Various avenues of research have been conducted about the impact of financial development on economic growth and environmental performance [10,26], and the econometric analysis based on theoretical justification has been widely applied to the field [27]. For example, Zhang et al. (2011) and Fang et al. (2020) found that whilst financial development has stimulated China’s economic growth, but this comes at the cost of a huge increase in carbon emissions [28,29]. On the other hand, Martinsson (2010) suggested financial development did not necessarily have a negative environmental impact [30]. He posited that bank-oriented and market-oriented development models had opposing effects on environmental performance. Whilst other scholars argued that finance development contributed to improving environmental performance [31], and the promoting effect varies in different regions [32].

Recently, China’s emerging DIF has developed rapidly supported by developments in big data, cloud computing, and artificial intelligence [33]. The spread of DIF is leading to dramatic changes in the structure and scale of agricultural carbon emissions [31]. Given China’s monumental carbon reduction goals, it is important to explore the impact of DIF on ACEP and propose some corresponding suggestions. Existing studies have pointed out that DIF has disrupted traditional finance in technological investment, market transactions, and environmental performance [11,28]. However, few studies have been conducted to explore the relationship between DIF and ACEP. Furthermore, due to the spatial agglomeration and regional differences of agricultural resources and production conditions, agricultural production and its carbon emissions may be affected not only by internal elements but also by surrounding factors [29]. Therefore, spatial autocorrelation should be considered into the impact of DIF on ACEP [34]. Traditional analysis methods, such as liner regression model, logistic regression model, and correlation analysis, ignore the spatial spillover effect of spatial elements [20,29]. This limitation may result in biased outcomes of global regression models [35]. Spatial regression models, including spatial lag model (SLM), spatial error model (SEM), and spatial Durbin model (SDM), can be used to accurately explore the spatial spillover effect of independent variables on dependent variables by considering the spatial autocorrelation [36]. Therefore, this method can provide more accurate and useful information regarding the impact of DIF on ACEP, which can help to better understand the relationship between financial development and agricultural carbon reduction in China.

Given the shortcomings of the existing literature, this paper attempts to examine whether DIF has a positive impact on ACEP and whether there is a spatial spillover effect in this impact. Specifically, based on 2011–2020 panel data from China, this paper applies a super slacks-based measure (Super SBM) model to measure ACEP from the perspective of the total factor productivity. Following this, a panel data model and a spatial regression model are employed to empirically analyze the impact of DIF on ACEP and its mechanism in China. The two key contributions that this paper offers in comparison to the previous literature can be summarized as follows. First, this paper fills a gap in the literature. Few studies have examined the impact of DIF on ACEP, which can increase awareness of the importance of DIF and its application in agriculture. Second, it takes spatial spillover effect into account and examines the spatial spillover effect of DIF on ACEP in the surrounding provinces. With consideration of the reality of China’s agricultural development, this paper selects suitable input-output indicators and applies the Super SBM model to accurately measure and analyze the evolution trend of ACEP in China. It is therefore of significance for the scientific formulation of agricultural carbon emission reduction policies in China. Following the introduction, Section 2 will introduce the methods and data. Section 3 reports the empirical results and discussion, and Section 4 summarizes the conclusion and implications of this paper.

## 2. Materials and Methods

### 2.1. Theoretical Framework

**Figure 1:**
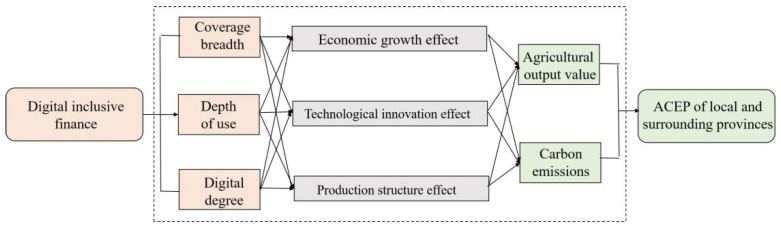
The mechanism of DIF on agricultural carbon emission performances.

As a new financial model, DIF can influence ACEP as follows. First, the development of DIF widens access to finance and reduces financing costs. DIF makes use of digital technologies to help farmers understand the role of financial products, stimulating farmers’ willingness to use financial services to affect the efficiency of agricultural green production [37]. Meanwhile, the expanding funding resources, collected from scattered financial resources at a lower cost, can provide more financing support for the development of eco-agriculture, circular agriculture, and smart agriculture [31]. Second, DIF can apply big data and cloud computing technology to produce a powerful function of matching capital supply and demand [38]. It can affect ACEP by guiding financial resources to green agricultural activities in key areas such as agricultural equipment, pollution prevention, and the cultivation of new subjects [31]. Third, by dispersing risk in a wide range, DIF can support agricultural technology innovation to affect ACEP. Technological innovation usually faces the risk that it cannot be implemented in the short term [34]. Owning to the spread of information technology to residents in remote areas, many scattered investors are attracted to the capital market. The considerable risks of agricultural technology innovation are effectively dispersed to a wide range, and ACEP may be improved through technology innovation (Figure 1).

With the increase in the scale of cross-regional flow of resources and the intensification of interregional competition for technological innovation resources, the development of local DIF would influence the surrounding ACEP. DIF can promote rural economic development by creating more employment opportunities, reducing financing costs for agricultural enterprises, and changing resident’ consumption patterns, which will attract more capital, enterprises and talents from neighboring areas to the local area [39]. On the other hand, advanced digital technology and financial services would also spillover to adjacent areas through knowledge and talent flow, the rural economic development in adjacent areas would be significantly affected, thereby influencing agricultural carbon emissions [40]. based on the above analysis, this study puts forward two hypothesis:

**Hypothesis** **1.** *The development of DIF will improve local ACEP.*

**Hypothesis** **2.** *The development of local DIF has significant spatial spillover effects.*

### 2.2. Calculation Method of ACEP

In the agricultural production process, inputs such as land, labor, capital, and technology, produce not only the agricultural products necessary for humans but also the carbon emission, which is called the undesired output. The SBM model, first proposed by Tone (2001), was more realistic as it takes into account the undesired output of the production process [18]. This model thus has been widely used to measure carbon performance, eco-efficiency, and energy efficiency [19,41]. Compared to traditional DEA models, the SBM model considers the slack variables and the efficiency in the presence of undesired outputs. Based on this, Tone (2002) then created a Super SBM model, which allowed the maximum value of the result to be greater than 1, thus making it possible to facilitate comparative rankings [42]. The ACEP can be measured by the following formula, which is as follows:

(1) $$\begin{array}{l} \rho =\min\frac{\frac{1}{m}\sum_{i=1}^{m}\frac{\bar{x_{i}}}{x_{i0}}}{\frac{1}{s_{1}+s_{2}}(\sum_{r=1}^{s_{1}}\frac{{\bar{y}}_{r}^{g}}{y_{r0}^{g}}+\sum_{q=1}^{s_{2}}\frac{{\bar{y}}_{j}^{b}}{y_{j0}^{b}})}\\ s.t.\{\begin{array}{c} x_{0}=X\lambda +S^{-},y_{0}^{g}=Y^{g}\lambda -S^{g},y_{0}^{b}=Y^{b}\lambda -S^{b}\\ \bar{x}\geq \sum_{j=1,\neq 0}^{n}\lambda_{j}x_{j},{\bar{y}}^{g}\leq \sum_{j=1,\neq 0}^{n}\lambda_{j}y_{j}^{g},{\bar{y}}^{b}\leq \sum_{j=1,\neq 0}^{n}\lambda_{j}y_{j}^{b}\\ \bar{x}\geq x_{0},{\bar{y}}^{g}\leq y_{0}^{g},{\bar{y}}^{b}\geq y_{0}^{b}\\ \sum_{j=1,\neq 0}^{n}\lambda_{j}=1,S^{-}\geq 0,S^{g}\geq 0,S^{b}\geq 0,{\bar{y}}^{g}\geq 0,\lambda \geq 0 \end{array} \end{array}$$

where *p* is the super efficiency value of the valued agricultural carbon emission, whose value can be larger than 1; *m*, *s*_1_, and *s*_2_ represent the number of input indicators, the number of desired output indicators, and the number of undesired output indicators, respectively; *x_i_*, *y^g^*, and *y^j^* are the value of inputs, desired output, and undesired output, respectively; $\bar{x_{i}}$, ${\bar{y}}_{r}^{g}$ and ${\bar{y}}_{j}^{b}$ represent the mean value of input, desired output, an undesired output, respectively; *λ* is the weight vectors; $S^{-}$, $S^{g}$ and $S^{b}$ are slack vectors corresponding to the abundance of inputs, scarcity of desirable output, and excess of undesirable output, respectively.

The estimated decision units are completely efficient when *p* > 1; otherwise, it is inefficient. In this paper, the expected output of agriculture was expressed by the agricultural output value (billion dollars) and the non-desired output was represented by the agricultural carbon emissions (million tons). Carbon emission is the major global climate change contributor and can reflect various pollutants in agricultural production [43]. According to the literature [3,41], agricultural inputs in this paper include land area, land force, agricultural machinery power, chemical fertilizer, pesticide, agricultural film, and irrigation (Table 1).

### 2.3. Agricultural Carbon Emission Measurement

Agricultural carbon emissions are derived from inputs and outputs in the agricultural production process. According to IPCC (2007) and Jiang et al. (2019), four main sources of these emissions are as follows [43,44]: The first is the use of agricultural inputs such as pesticides, chemical fertilizers, and agricultural film. The second is tillage, which results in a large amount of organic carbon entering the air. The third is the energy consumed in the process of agricultural irrigation. The fourth is the feeding of main livestock. The measurement formula of agricultural carbon emissions is as follows:

(2) $$ACE=\sum AC_{i}=\sum S_{i}\times \varepsilon_{i}$$

where *ACE* is the total agricultural carbon emissions, *AC_i_* is the emissions for the *i*-th agricultural carbon source, *S_i_* is the amount of the *i*-th agricultural carbon source, and is the emission coefficient of the *i*-th agricultural carbon source. The emission coefficients of various agricultural carbon sources are described in Table 2.

### 2.4. Spatial Econometric Model

#### 2.4.1. Basic Model

According to the primary hypotheses put forward in this study, a panel regression model is used to quantify the impact of DIF on ACEP. Panel regression data refers to pool data collected from both cross-sections and time. Supported by large samples, panel data provides a greater level of insight into the dynamic behavior of individuals. Subsequently, it overcomes the problem of absent variables, thus improving the accuracy of results [46]. The specific model is as follows:

(3) $$\ln acep_{i,t}=\beta_{0}+\sum_{n=1}^{3}\beta_{n}\ln dif_{i,t}+\sum_{m=1}^{5}\beta_{m}\ln ctl_{i,t}+\lambda_{i}+\eta_{t}+\varepsilon_{i,t}$$

where *i* is the provincial administrative unit, *t* represents time, *β* is the estimated parameter, *λ* represents individual effects, *η* represents time effects, *ε* represents the random disturbance term of normal distribution. *lnacep*_*i*,*t*_ is the logarithm of ACEP; *lndif*_*i*,*t*_ is the logarithm of digital inclusive finance indicators; and *lnctl*_*i*,*t*_ is the logarithm of control variables.

#### 2.4.2. Spatial Autocorrelation Analysis

This study attempts to employ the Moran’s I index to test whether the ACEP among the provinces has spatial effects. Moran’s I index includes the Global Moran’s I iindex and Local Indicator of Spatial Association (LISA) [47]. The specific formula for Global Moran’s I index is expressed as follows:

(4) $$I=\frac{n}{\sum_{i=1}^{n}\sum_{j=1}^{n}w_{ij}}•\frac{\sum_{i=1}^{n}\sum_{j=1}^{n}w_{ij}•(m_{i}-\bar{m})(m_{j}-\bar{m})}{\sum_{i=1}^{n}{\left(m_{i}-\bar{m}\right)}^{2}}$$

where *I* is the Global Moran’s index, *n* is the number of spatial units, *w_ij_* is the geographic distance weight matrix, *m_i_* and *m_j_* are the values of ACEP in unit *i* and *j*, respectively, $\bar{m}$ is the average value of ACEP for the entire region. The range of Moran’s I is from −1 to 1. The larger the value of Moran’s I, the higher the spatial correlation between units. When the value of Moran’s I index is close to −1, indicating that there is a significant negative spatial correlation between units. If the value of Moran’s I is close to zero, indicating that the value of ACEP is random distributed. Additionally, to identify the local spatial agglomeration of ACEP, the LISA statistic is expressed as follows:

(5) $$I_{i}=\frac{n(m_{i}-\bar{m})}{\sum_{i=1}^{n}{\left(m_{i}-\bar{m}\right)}^{2}}•\sum_{i=1,j\neq 1}^{n}w_{ij}•(m_{j}-\bar{m})$$

where *I_i_* is the local Moran’s I index, reflecting the spatial correlation degree between unit *i* and adjacent unit *j*. The definitions of the other variables are the same as those of Formula (4). When the value of *I_i_* is greater than 0, indicating that the value of ACEP in unit *i* is similar to adjacent unit *j*. When the value of *I_i_* is less than 0, indicating that the value of ACEP in unit *i* is different from adjacent unit *j*.

#### 2.4.3. Spatial Regression Model

Traditional panel regression model ignores the possible spatial dependence of variables. To exploring the possible spatial spillover effect of DIF on ACEP, three widely used patial regression models, including spatial lag model (SLM) (Fornula (6)), spatial error model (SEM) (Fornula (7)), and spatial Durbin model (SDM) (Fornula (8)) are constructed in this study [35]. These models are based on different types of spatial effects. The specific formulas are as follows:

(6) $$\ln acep_{i,t}=\beta_{0}+\sum_{n=1}^{3}\beta_{n}•\ln dif_{i,t}+\sum_{m=1}^{5}\beta_{m}•\ln ctl_{i,t}+\rho •\sum_{j=1}^{31}w_{ij}•\ln acep_{j,t}+\lambda_{i}+\eta_{t}+\varepsilon_{i,t}$$

(7) $$\ln acep_{i,t}=\beta_{0}+\sum_{n=1}^{3}\beta_{n}•\ln dif_{i,t}+\sum_{m=1}^{5}\beta_{m}•\ln ctl_{i,t}+\lambda_{i}+\eta_{t}+\varepsilon_{i,t}+\delta •\sum_{j=1}^{31}w_{ij}•v_{j,t}$$

(8) $$\begin{array}{ll} \ln acep_{i,t}= & \beta_{0}+\sum_{n=1}^{3}\beta_{n}•\ln dcf_{i,t}+\sum_{m=1}^{5}\beta_{m}•\ln ctl_{i,t}+\rho •\sum_{j=1}^{31}w_{ij}•\ln acep_{j,t}\\ & +\theta •\sum_{j=1}^{31}w_{ij}•\ln dcf_{j,t}+\omega •\sum_{j=1}^{31}w_{ij}•\ln ctl_{j,t}+\lambda_{i}+\eta_{t}+\varepsilon_{i,t} \end{array}$$

where *w_ij_* is the geographic distance weight matrix, *ρ* represents the spatial autoregression coefficient, which can measure the spatial spillover effect of the explained variable in surrounding units on this unit, *θ* and *w* represent the coefficients of the spatial lag variables respectively, reflecting the spatial spillover effect of the explanatory variables in surrounding units on the explained variable, respectively. *δ* is the coeffcient of spatial component errors. The definitions of the other variables are the same as those of Formula (3). According to the litature [48], several test including the Lagrange multiplier (LM) and robust LM tests are conducted to choose the suitable algorithm for spatial regression.

According to Lesage and Pace (2009), the spatial regression model partial differential method can be used to decompose the effect of the coefficients [49]. Thus, the direct, indirect, and total effect estimates are employed to interpret the model. In this study, direct effect refers to the impact of the development of DIF on local ACEP, including the spatial feedback effect. The indirect effect refers to the impact of the development of DIF on ACEP in the adjacent areas, which is considered as the spatial spillover effect of DIF on ACEP. The specific process formula can refer to Zhong et al. (2022) [34].

### 2.5. Variables Selection and Data Sources

#### 2.5.1. Explained Variable and Core Explanatory Variable

This paper uses the measurement result of ACEP as the explained variable. Meanwhile, DIF is chosen as the core explaining variable. In this paper, the DIF index, obtained from “The Peking University Digital Financial Inclusion Index of China”, is used as the proxy variable. The index is synthesized based on financial service data provided by Ant Financial at the provincial, city, and county levels in China [50]. The digital inclusive financial index includes 3 sub-dimensions: the coverage breadth, the depth of use, and the digitalization degree. The coverage breadth mainly includes three indicators: the number of Alipay accounts, the proportion of Alipay account-bound users, and the number of Alipay account-bound cards, which reflect numbers Financial coverage; the depth of use covers payment, credit, insurance, investment, and monetary funds and other services, reflecting the increase in the variety and availability of digital financial instruments; the digitalization degree includes mobile, affordability and facilitation, which can reflect the degree of integration and inclusiveness of digital finance and digital technology. The index has been widely applied to the field of China’s DIF [9,26], and has considerable representativeness and reliability. This paper uses these three sub-indicators as explanatory variables to further explore the impact of different dimensions of digital finance on ACEP.

#### 2.5.2. Control Variables

Referring to the previous research, some other influencing factors were selected as control variables. According to Wu et al. (2020) and Gao et al. (2021) [51,52], the value of GDP per capita and and the urbanization rate of the resident population are used to reflect the regional socio-economic development level. The improvement of the industrialization level can provide material conditions and product markets for agriculture, as well as technical support, promoting agricultural economic development. Thus the ratio of output value of secondary industry to GDP is selected to measure the level of regional industrial development. The higher the opening-up level, the easier it is to learn from advanced foreign technology and management experience, which can promote the green development of agriculture [34]. Therefore, the proportion of foreign trade volume of agricultural products in total agricultural output value is employed to indicate the level of opening up. Considering that agricultural disasters lead to higher losses of agricultural inputs, the proportion of crop disaster areas in crop sown area is included in the control variables. Additionally, rural economic development is closely related to agricultural production activities. The increase in farmers’ income results in changes in the structure and model of agricultural production. Thus the per capita disposable income of rural households is used to measure.

#### 2.5.3. Data Sources and Descriptive Statistics

The research sample in this study covers 31 provinces and cities in China. The availability of data in the balanced provincial panel data from 2011 to 2020 is broad and given the validity of the data, it is employed for empirical research. In the sample, Eastern China includes Liaoning, Hebei, Beijing, Tianjin, Shandong, Jiangsu, Shanghai, Zhejiang, Fujian, Guangdong, and Hainan; Central China consists of Jilin, Heilongjiang, Neimenggu, Shanxi, Henan, Anhui, Jiangxi, Hubei, and Hunan, Guangxi; while Western China consists of Chongqing, Sichuan, Guizhou, Yunnan, Shaanxi, Gansu, Qinghai, Ningxia, Xinjiang, and Xizang. The above data are obtained from the China Statistical Yearbook (2012–2021), China Rural Statistical Yearbook (2012–2021), Chinese Agricultural Statistical Yearbook (2012–2021), and the Chinese Land Statistical Yearbook (2012–2021). Table 3 shows the descriptive results of the variables calculated by SPSS. 22.0.

## 3. Results and Discussion

### 3.1. Spatial-Temporal Changes in ACEP

**Figure 2:**
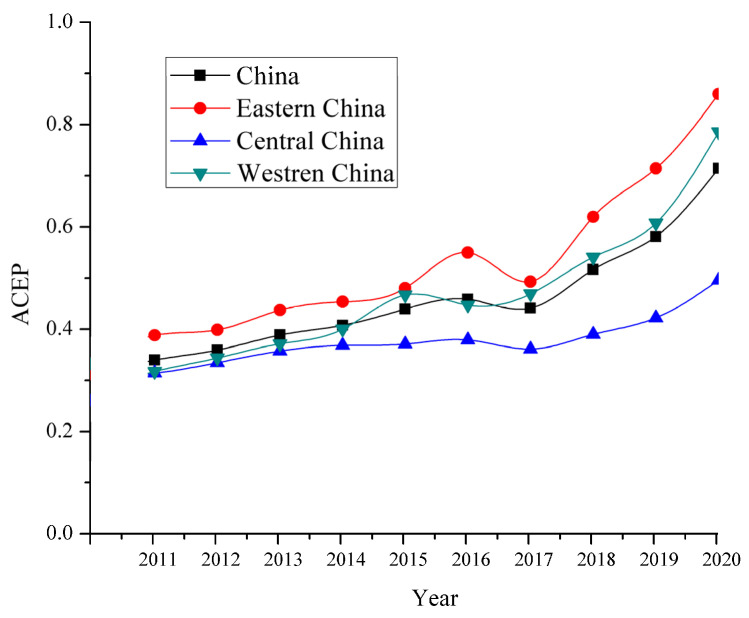
The trend of ACEP in three geographical regions of China between 2011 and 2020.

**Figure 3:**
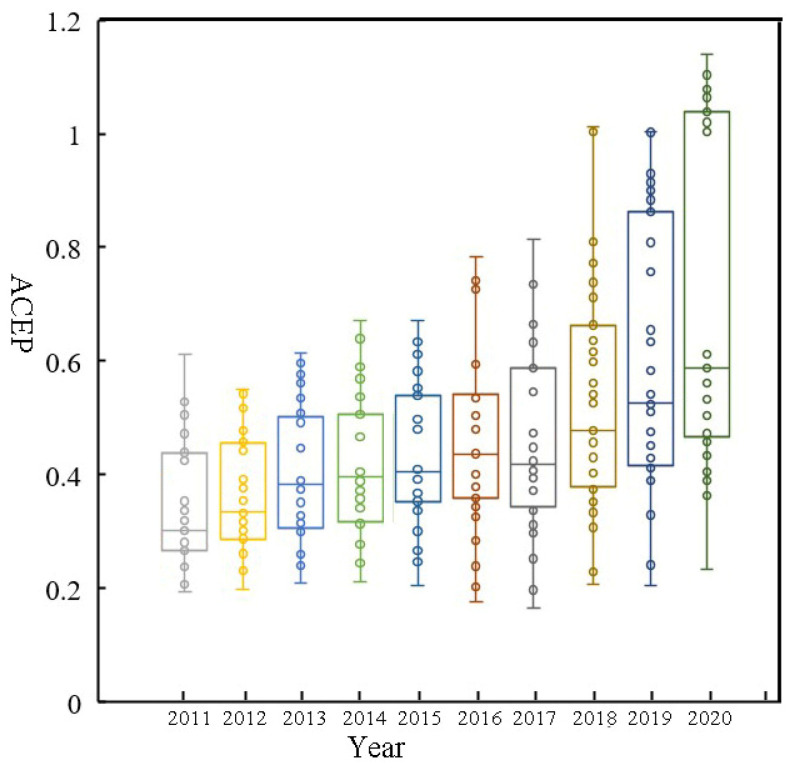
Box diagram of ACEP by the province between 2011 and 2020.

Figure 2 shows the average level of ACEP in the country and three regions (east, central and west) between 2011 and 2020. China’s ACEP exhibited a fluctuating growth trend from 0.32 in 2011 to 0.71 in 2020. In particular, the growth rate of ACEP further accelerated after 2017. The reason for this phenomenon is that, since 2015, the Chinese government has proposed an agricultural green development strategy, and attempted to achieve zero growth in chemical fertilizers and pesticides [53]. Meanwhile, local governments have formulated corresponding measures to protect the agricultural environment [54]. In different regions, the average ACEP in eastern, central, and western regions generally showed a trend of increasing, from 0.39, 0.31, and 0.30 in 2011 to 0.86, 0.50, and 0.79 in 2020, respectively. The ACEP in Eastern China was the highest, followed by Western China, and the lowest ACEP occurred in Central China. The box plot illustrates the discrete distributions of ACEP values in China’s provinces from 2011 to 2020 (Figure 3). It can be seen that the average value of ACEP developed from a concentration of low values to a spread towards the two ends and a concentration of medium values, indicating that the regional differences in ACEP between provinces widened over the study period.

**Figure 4:**
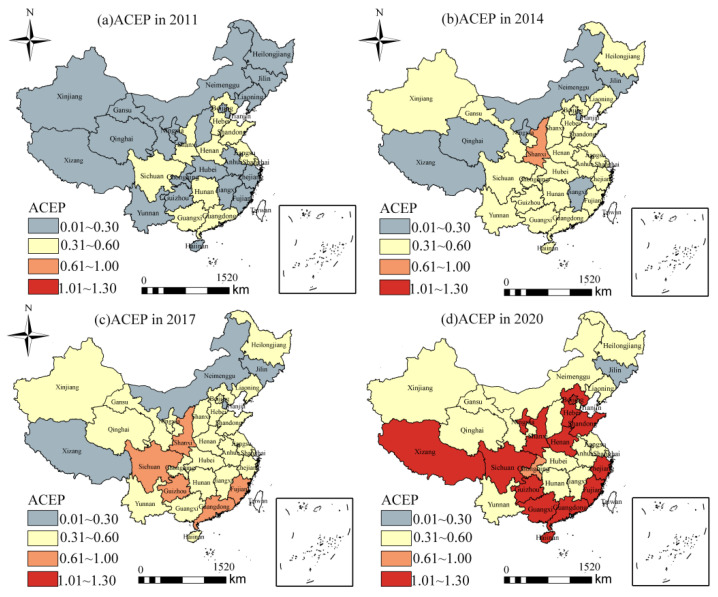
Spatial distribution of ACEP in each province between 2011 and 2020.

The spatial distribution of ACEP was generally high in the eastern and southern regions and low in the northern and western regions of China (Figure 4). In 2011, 67% of provinces had ACEP values below 0.30, mainly located in western, northern, and southeastern China. The ACEP values of the other provinces were in the range of 0.30–0.60, and high-value areas were distributed in the Huang-Huai-Hai plain and the Pearl River Delta, indicating that there was a large potential for technological advancement and low carbon emission reduction in China’s agricultural development process. Between 2011 and 2020, the vast majority of provinces showed a significant improvement in ACEP over the study period, especially between 2017 and 2020. In 2020, fourteen provinces in China had maintained efficient values (value > 1) for ACEP, with Hainan having the highest value (1.113). These provinces are mainly located in the coastal regions and the Yellow River basin. These are relatively developed regions or important major agricultural production areas, where local governments have access to land, labor, capital, and technology to promote agricultural production efficiency [52]. However, the central and northern provinces were found to have lower values of ACEP, with Jilin having the lowest value (0.23). Some of these provinces are large agricultural provinces, which indicates that these provinces still have input and large carbon emissions.

### 3.2. Benchmark Regression Analysis

#### 3.2.1. The Correlation between DIF and ACEP

**Figure 5:**
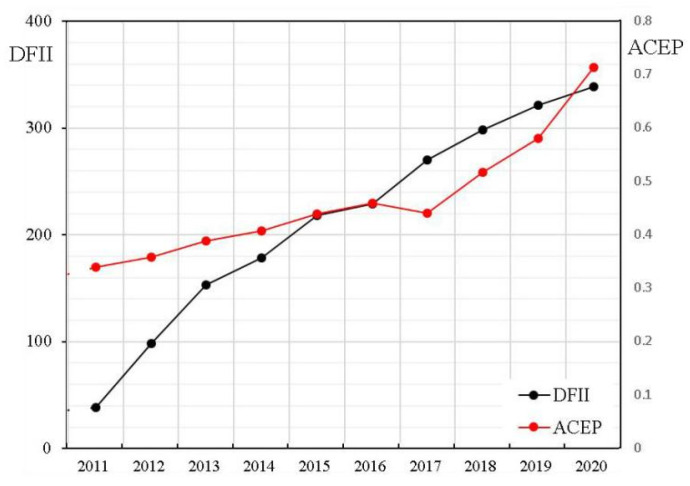
The changing trend lines of DFII and ACEP.

As shown in Figure 5, the digital inclusive financial level in China showed rapid growth between 2011 and 2020, with its growth rate accelerating after 2013. Specifically, the digital inclusive financial index increased from 30.19 in 2011 to 339.01 in 2020, with an average annual growth rate of 98.02%. At the same time, ACEP also displayed a significant upward trend. Relying on the Internet, big data, cloud computing, and other advanced information technologies, DIF can broaden the coverage of traditional finance and effectively promote the financial accessibility of farmers and disadvantaged groups in remote areas [55]. This advantage helps to alleviate the financing difficulties for farmers and increase access to finance for green agricultural projects [7]. As a result, ACEP had an obvious growth trend consistent with the digital inclusive financial level.

#### 3.2.2. Basic Regression Analysis

Four different specifications of panel data regressions are used to measure DIF’s impact on ACEP (Table 4). These include the ordinary least square (OLS), the pooled ordinary least squares (POOL), the fixed effect (FE), and the random effect (RE). The Hausman test indicates that the FE-effects model is more suitable for econometric analysis. According to Table 4, the coefficient of DIFI was positive and significant at the 5% level. The data shows that for every percentage point increase in the level of DIF, there was a corresponding 0.207 percentage point increase in the efficiency of agricultural carbon emissions. This indicates that, within China, DIF has a positive impact on ACEP. This result confirms the finding of He et al. (2019) [56], who indicated that digital inclusive financial development had a positive effect on agricltural green total factor productivity. The spread of DIF alleviates the financial constraints faced in rural areas. It can simplify the complex and bureaucratic business processes of traditional finance and promote innovation in sectors that have often lacked it. Over the past 10 years, the Chinese government has issued a series of documents that strongly advocate the spread of digital finance in agriculture and within rural areas. The documents emphasize the role green finance plays in promoting solutions to the emissions problem within agriculture [53]. Meanwhile, digital currency, third-party payment, and online loans are gaining popularity in rural China [57]. These policies and measures have enhanced the impact of DIF on agricultural carbon reduction.

Moving to consider the influences of control variables, the coefficient of PDC was −0.384, this was at the 5% level and therefore significant. This indicated that a 1% increase in the PDC would reduce ACEP by 0.384%. This regression result is consistent with the findings of Fang et al. (2021) [7]. The main reason for this finding is that losses due to natural disasters reduce yields and reduce the efficiency of agricultural carbon emissions. The coefficient of PIR was 0.621 at the 5% significance level, implying that PIR played a significant positive role in promoting ACEP. A likely cause is that as per capita income rises, farmers tend to upgrade their agricultural input, purchasing higher quality seeds. They also invest in improving agricultural infrastructures, such as irrigation and advanced machinery. These are all factors conducive to improving the efficiency of agricultural carbon emissions [58]. The coefficients of other variables, including PGDP, URP and RSI, were not statistically significant, indicating that these factors had no significant impact on ACEP.

#### 3.2.3. The Influences of Different Dimensions of DIF on ACEP

According to the optimal model obtained by the above analysis, this study uses the FE_model to further reveal the influence mechanism of DIF on ACEP. Table 5 presents the estimated results for the three digital inclusive financial indices. The results show that the coefficient of CB was positive at the 1% significance level, indicating that there was a positive relationship between financial coverage breadth and ACEP, and this relationship was significant. The findings show that for every one percentage point increase in the CB, ACEP would increase by 0.459%. The expansion of digital financial coverage breadth allows digital access to financial information whilst transactions can take place via the online platform. The spatial-temporal constraints of traditional financial institutions are thereby overcome. This is especially pertinent and extending the coverage of users to rural areas where access to traditional institutions is limited. On the other hand, the services of DIF target micro, small and rural enterprises, whose needs and particular circumstances are not serviced by traditional financial services. The solutions provided by DIF extend the coverage of users for financial services and alleviate financial exclusion in rural areas [59]. The coefficient of DU was 0.24 at a 5% significant level, indicating that the increased depth of use of DIF in China contributes to a more efficient agriculture and thereby reduces carbon emissions. DU reflects the variety and availability of digital financial instruments. Its improvement can increase the use frequency of financial products, this offers greater flexibility to those working in the industry and allows them to meet the challenges of reducing emissions. However, the results revealed that DD had no significant correlation with ACEP. This is likely due to the lack of modern digital integration within China’s traditional financial institutions, resulting in high financial services costs and high thresholds. These factors prove to be a hindrance to the improvement of ACEP.

### 3.3. Spatial Regression Analysis

#### 3.3.1. Spatial Autocorelation Analysis of ACEP

As shown in Table 6, the Global Moran’s index for ACEP from 2011 to 2020 were all greater than 0, at *p* < 0.05, indicating that ACEP in China exhibited a significant positive spatial autocorrelation. This phenomenon may occur because agricultural production is determined by natural endowment. Adjacent regions have similar agricultural resources and production patterns, resulting in the spatial aglomeration of ACEP [22]. During 2011 and 2020, the Global Moran’s I index for ACEP showed a decreasing trend, indicating that the spatial agglomeration of ACEP in China continued to weaken. This is mainly because that agricultural production has been deeply influenced by urbanization and industrialization during the study period. Numerous agricultural labor population and land resources flowed into urban areas, resulting in significant changes in regional agricultural production mode. Meanwile, owing to the improvement of agricultural technology, agricultural total factor efficiency in different regions has been increased, leading to the weakening of spatial aglomeration of ACEP [11]. 

**Figure 6:**
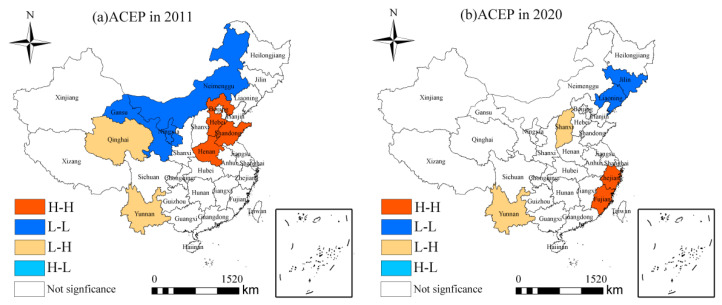
LISA map of ACEP in 2011 and 2020.

Figure 6 exhibits the spatial agglomeration results of ACEP in 2011 and 2020. The high spatial agglomeration areas of ACEP in 2011 were mainly distributed in Huang-Huai-Hai Plain, including Hebei, Shandong, and Henan Province. These areas are the main agricultural producing areas in China. The low spatial agglomeration areas of ACEP in 2011 were concentrated in northern China, including Neimenggu, Gansu and Ningxia, which are the main pastoral regions in China. During 2011 and 2020, the number of provinces with high spatial agglomeration of ACEP decreased to 2, and transfered to China’s eastern coastal areas, including Zhejiang and Fujian. This can be explained that these areas could lead the development of ACEP in surrounding areas through advanced agricultural technology cooperation and exchange [26]. Meanwhile, the low spatial agglomeration areas transferred to the notheastern China, including Jilin and Liaoning Province. These findings further confirmed the previous conclusion that China’s ACEP displayed a significant positive spatial agglomeration.

#### 3.3.2. Selection of Spatial Model

To determine which spatial regression model is more suitable for the estimation. The tests of LM, Wald, LR, Hausman, and fixed effects are conducted. As shown in Table 7, the values of LM-LAG, Robust LM-LAG, LM-ERR, and Robust LM-ERR all passed the significant test, at *p* < 0.01, indicating that spatial regression models were more suitable for analyzing the impact of DIF on ACEP. Furthermore, the test results of Wald-SAR, Wald-SEM, LR-SAR, and LR-SEM also passed the significant test, indicating that SDM should be used to quantify the spatial spillover effect of DIF on ACEP [34]. In addition, the Hausman result displayed that fixed effect model was more suitable than random effect model. Therefore, the fixed effect SDM model was used to analyze the impact of DIF on ACEP by considering the spatial spillover effect in this study.

#### 3.3.3. Analysis of SDM Results

The SDM results of the spatial effect of DIF on ACEP in China are shown in Table 8. The value of spatial autocorrelation coefficient *ρ* was significantly positive, indicating that ACEP was significantly affected by surrounding areas. It can be seen that the coefficient of DIFI had positive impacts on local ACEP, further verifying the role of the development of DIF in improving ACEP. Meanwhile, it shows that W*DIFI was significantly negative, indicating that the development of DIF could also improve ACEP in the surrounding provinces. This result is consistent with Liu et al. (2022), who showed that the digital technology could reduce carbon emissions in the surrounding regions [22]. This is mainly because that the development of local DIF can promote the inter-provincial flow of agricultural green production technologies and green funds, thereby promoting the improvement of ACEP in surrounding areas [11]. Furthermore, the coefficients of W*RSI and W*PDC were negative, indicating that the secondary industry to GDP and the proportion of crop disaster areas in crop sown area in local areas had negative impacts on ACEP in the surrounding regions. Geographically adjacent areas are prone to the same natural disasters, so when the natural disaster area increases, agricultural production in adjacent areas will also be affected [34]. It also shows that the per capita disposable income of rural households had a positive impact on ACEP in the surrounding regions.

Table 9 displays the results of direct effect and indirect effect of DIF. It can been seen that the coefficients of DIFI were positive for both direct effect and indirect effect, indicating that the development of DIF had positive impacts on both the local and surrounding ACEP. The coefficient of DIF with direct effect was greater than that with indirect effect, indicating that the impact of DIF on local ACEP was greater than that on surrounding ACEP. In terms of the control variables, the coefficient of PDC for indirect effect was −0.182, which was larger than that for direct effect, indicating that the negative impact of PDC on local ACEP was greater than that on surrounding ACEP. The coefficient of PIR for direct effect was 0.329, which was larger than that for indirect effect, indicating that the positive impact of PIR on local ACEP was greater than that on surrounding ACEP.

## 4. Conclusions

Digital inclusive financial development has broken through the service boundaries of conventional finance and effectively matched the capital supply and demand [37]. It has become an important driver of low-carbon agricultural development. This paper calculates the agricultural carbon emissions and employs the Super SBM model to measure ACEP. A panel regression model and a spatial Durbin model are used to systematically examine the impact of DIF on ACEP. The main findings are as follows. (1) China’s average ACEP increased from 0.32 in 2009 to 0.71 in 2020, displaying a fluctuating growth trend. The main concentration areas with high-value ACEP transformed from the Huang–Huai–Hai plain and Pearl River Delta to the coastal regions and the Yellow River basin, and the differences between provinces gradually increased. (2) The regression results indicate that DIF had a significant positive impact on ACEP. The coverage breadth and depth of use of DIF could significantly improve ACEP, but the digital degree was found to have no significant effect. (3) The development of DIF can not only improve local ACEP, but also improve ACEP in the surrounding provinces through the spatial spillover effect.

Several of these important policy implications become apparent given these conclusions. First, the Chinese government should continue to support digital inclusive financial development in rural areas. Specifically, the construction of rural digital infrastructure, such as network base stations and home broadband, should be a push for greater access to smartphones, tablets, and computers. Both coverage and hardware need to be accessible and affordable. In conjunction with this, financial literacy in rural areas must be increased. By increasing knowledge of available financial services and moderately reducing the broadband cost for those most closely connected to the agriculture industry, the depth of use of DIF can be improved. Additionally, accelerating the deep integration of traditional finance and digital technology is also conducive to improving the digitization degree of DIF. Second, green development is the core of China’s ecological civilization construction. To promote green development, it oughts to not only guide the inflow of various resource into the green devlopment field, but also reflect the ecological concept in the mode of production organization. This requires the government to give play to the role of financial policies and financial instruments in guiding and structural adjustment. Specifically, the government should redirect funds into agricultural technology innovation activities. The goal should be to promote technological progress and diffusion rather than scale expansion. More importantly, the existence of spatial spillover effect of DIF on ACEP indicates that the digital finance development policies formulated by local government will have significant impacts on the surrounding areas. Therefore, when formulating policies related to agricultural carbon emission reduction and digital financial development, local managers ought to start from the macro-control scale, then break administrative boundary barriers and strengthen regional cooperation. For example, the development of digital finance can formulate relevant planning policies by taking urban agglomerations as a whole, which can promote the agglomeration effect of digital finance industry and strengthen the collaborative governance of agricultural carbon emission reduction.

Although some progress has been made, this study has a few limitations. First, the data sample of this paper only includes China, a developing country. The development of DIF and its application in agricultural production activities in developing countries may be different from those in developed countries. Future research needs to add samples obtained from other regions, including Eastern Asia, Europe, and North America. Second, this paper conducts empirical analysis from a macro perspective but fails to do so from a micro perspective. Future research could obtain first-hand data on DIF at the rural household-scale through field surveys, and analyze the impact of DIF on low-carbon agriculture from the perspective of peasant households.

## Figures and Tables

**Table 1:** Input and output variables for measuring ACEP.

First Level Index	Second Level Index	Variable Description
Input variables	Land area	The planting area of crops (1000 hectares)
	Labor force	The number of employees on the farm (10,000 people)
	Agricultural machinery power	Total power of agricultural machinery (10,000 kilowatts)
	Chemical fertilizer	Total fertilizers consumption (10,000 tons)
	Pesticide	Pesticides usage (10,000 tons)
	Agricultural film	Agricultural film consumption (ton)
	Irrigation	Amount of water used for Irrigation (10^8^ m^3^)
Output indicators	The total output value of the farm	Constant price in 2011 (1 × 10^8^ yuan)
Unexpected output	Agricultural carbon emissions	Measurement based on agricultural input and output elements (1 × 10^3^ ton)

**Table 2:** Carbon emission coefficients of different input elements in the agricultural production process.

Carbon Source	Selected Metrics	Carbon Emission Coefficients	Sources
Chemical fertilizer	Total fertilizers consumption (10,000 tons)	0.8965 kg kg^−1^	Oak Ridge National Laboratory, ORNL
Pesticides	The amount of pesticide used (10,000 tons)	4.9341 kg kg^−1^	Oak Ridge National Laboratory, ORNL
Agricultural film	The amount of agricultural plastic film (ton)	5.18 kg kg^−1^	Institute of Resources, Ecosystem, and Environment of Agriculture, IREEA
Agricultural machinery	The amount of agricultural diesel used (10,000 tons)	0.5927 kg kg^−1^	IPCC (2007)
Agricultural ploughing	The total planting area of crops (1000 hectares)	312.6 kg km^−2^	Wu et al. (2007) [5]
Agricultural irrigation	Effective irrigation area (hectares)	20.476 kg/hm^−2^	Dubey and Lal (2009) [45]
Pigs	The number of pigs at end of the year	34.0910 kg/ (each year)	IPCC (2007)
Cattle	The number of cattle at end of the year	415.91 kg/ (each year)	IPCC (2007)
Sheep	The number of sheep at end of the year	35.1819 kg/ (each year)	IPCC (2007)

**Table 3:** Descriptive statistics of the variables.

Variable	Name	Abbreviation	Obs.	Mean	Max	Min	C. V.
Explained variable	Agricultural carbon emission performance	ACEP	372	0.45	1.14	0.15	0.47
Core explaining variables	DIF index	DIFI	372	185.61	431.93	6.22	0.60
	Coverage breadth	CB	372	168.53	397.00	1.46	0.65
	Depth of use	DU	372	182.55	488.68	2.76	0.60
	Digitalization degree	DD	372	248.32	462.23	3.58	0.57
Control variables	Value of GDP per capita	PGDP	372	51,586.28	164,889.00	10,309.00	0.53
	Urbanization rate of the resident population	URP	372	0.57	0.89	0.23	0.24
	Ratio of the secondary industry to GDP	RSI	372	0.44	0.59	0.16	0.20
	Proportion of foreign trade volume of agricultural Products in total agricultural output value	PFA	372	0.36	0.53	0.01	0.31
	Proportion of crop disaster areas in crop sown area	PDC	372	0.14	0.48	0.02	0.35
	Per capita disposable income of rural households	PIR	372	11,394.52	34,911.30	2980.10	0.50

**Table 4:** Basic regression model results.

Variables	(1) OLS_Model	(2) POOL_Model	(3) FE_Model	(4) RE_Model
DIFI	0.587 *** (6.298)	0.587 *** (3.512)	0.207 ** (−1.778)	0.340 ** (2.299)
PGDP	−0.004 (−0.029)	−0.004 (−0.017)	0.176 (1.050)	−0.037 (−0.237)
URP	−0.300 *** (−2.812)	−0.300 *** (−2.706)	0.027 (−0.293)	−0.074 (−0.855)
RSI	0.036 (0.674)	0.036 (0.490)	−0.064 (−0.634)	0.040 (0.520)
PFA	0.271 ** (7.878)	0.271 ** (4.887)	0.019 (0.113)	0.098 (1.103)
PDC	−0.190 ** (−4.683)	−0.190 * (−2.354)	−0.384 ** (−2.968)	−0.321 ** (−3.317)
PIR	0.066 (0.387)	0.066 (0.197)	0.621 ** (3.097)	0.336 ** (1.363)
R^2^	0.434	0.434	0.667	0.356
R^2^ (adj)	0.423	0.477	0.547	0.514
Obs	372	372	372	372
F statistics	52.565 ***	37.25 ***	23.926 ***	165.299 ***

Notes: t statistics are in parentheses. * *p* < 0.1, ** *p* <0.05, *** *p* < 0.01.

**Table 5:** Estimation results for the three financial development indices.

Variables	FE_Model Results		
CB	0.459 ***		
DU		0.240 **	
DD			0.03
PGDP	0.015	−0.050	−0.073
URP	−0.135 *	0.001	0.022
RSI	0.068	0.020	−0.001
PFA	0.145 *	0.030	−0.021
PDC	−0.316 **	−0.336 **	−0.334 **
PIR	0.195	0.448 *	0.697 **
R^2^	0.397	0.285	0.197
R^2^ (adj)	0.523	0.518	0.521
Obs	372	372	372
F statistics	χ^2^(7) = 244.427 ***	χ^2^(7) = 131.237 ***	χ^2^(7) = 114.260 ***

Notes: t statistics are in parentheses. * *p* < 0.1, ** *p* < 0.05, *** *p* < 0.01.

**Table 6:** The results of Global Moran’s I index for ACEP from 2009 to 2020.

Year	Moran’s I Index	*p*-Value
2009	0.1835	0.021
2010	0.1716	0.025
2011	0.1647	0.035
2012	0.1945	0.038
2013	0.1694	0.040
2014	0.1580	0.045
2015	0.1548	0.043
2016	0.1331	0.043
2017	0.1249	0.051
2018	0.1252	0.039
2019	0.1187	0.045

**Table 7:** The test results of LM, Wald, LR, and Hausman.

Test	Value	Test	Value
LM-LAG	254.6732 ***	Wald-SAR	63.2178 ***
Robust LM-LAG	14.3760 ***	Wald-SEM	52.5764 ***
LM-ERR	169.5426 ***	LR-SAR	60.8957 ***
Robust LM-ERR	8.5624 ***	LR-SEM	55.3210 ***
Hausman	6.8932 **		

Note: ** *p* < 0.05, *** *p* < 0.01.

**Table 8:** The estimated results of SDM.

Variables	SDM	Variables	SDM
DIFI	0.198 ***	W*DIFI	0.107 ***
PGDP	0.143	W*PGDP	0.186
URP	0.036	W*URP	0.091
RSI	−0.124	W*RSI	−0.057 **
PFA	−0.008	W*PFA	0.012
PDC	−0.298 ***	W*PDC	−0.122 ***
PIR	0.564 ***	W*PIR	0.284 ***
ρ	0.289 ***	Log−likelihood	864.682

Note: ** *p* < 0.05, *** *p* < 0.01.

**Table 9:** Direct and indirect effects of DIF on ACEP.

Variables	Direct Effect	Indirect Effect	Total Effect
DIFI	0.437 ***	0.136 **	0.573 ***
PGDP	0.214 *	0.045	0.259
URP	0.143	0.015	0.158
RSI	−0.218	0.167 *	−0.051
PFA	−0.106	0.112	0.006
PDC	−0.231 ***	−0.182 **	−0.413 ***
PIR	0.329 ***	0.267 ***	0.596 ***

Note: * *p* < 0.1, ** *p* < 0.05, *** *p* < 0.01.

## Data Availability

The data presented in this paper are available on request from the corresponding author.
